# Impact of playing position on ACL injury characteristics, risk and career outcomes in football—An analysis of 742 cases of the anterior cruciate ligament registry in German football

**DOI:** 10.1002/ksa.70566

**Published:** 2026-08-03

**Authors:** Shoichi Hasegawa, Werner Krutsch, Hidejuki Koga, Volker Alt, Dominik Szymski, Lorenz Huber

**Affiliations:** ^1^ Department of Joint Surgery and Sports Medicine Graduate School of Medical and Dental Sciences, Institute of Science Tokyo Tokyo Japan; ^2^ Sporthopaedicum Straubing Straubing Germany; ^3^ Department of Trauma Surgery University Medical Centre Regensburg Regensburg Germany; ^4^ FIFA Medical Centre of Excellence University Medical Centre Regensburg Regensburg Germany; ^5^ SportDocs Franken Nueremberg Germany

**Keywords:** epidemiology, football, injury, knee, sports

## Abstract

**Purpuse:**

To evaluate how playing position affects anterior cruciate ligament (ACL) injury features, incidence rates and career outcomes across competition levels in male football.

**Methods:**

Using the prospectively collected ‘ACL Registry in German Football’, we analysed six seasons (2016–2022) across professional (first to third leagues), semi‐professional (fourth to sixth) and amateur (seventh) levels. In professional players, return‐to‐performance over five seasons was evaluated with linear mixed‐effects models, and career outcomes within three seasons after injury were assessed. Injury mechanisms were examined across all levels. Outcomes were stratified by position (goalkeeper [GK], defender [DF], midfielder [MF], forward [FW]).

**Results:**

A total of 742 ACL injuries were analysed (professional *n* = 172, semi‐professional *n* = 316, amateur *n* = 254). Among professionals, FWs showed the highest rate of a drop in competitive level (47.5%), and GKs a numerically higher rate (54.5%); however, the GK comparison was not statistically significant after correction for multiple testing and should be regarded as exploratory. Mixed‐effects models for matches and minutes played showed significant main effects for time and position (all *p* < 0.001) but no significant interaction (*p* = 0.064 matches; *p* = 0.105 min). Match‐related incidence was highest in DFs (0.397/1000 player‐hours) and MFs (0.370). Injury mechanisms varied by level; direct contact accounted for 66.7% and 46.2% in professional FWs and DFs, respectively, whereas non‐contact injuries predominated across all positions in amateur players.

**Conclusions:**

The characteristics and outcomes of ACL injuries may vary by position. Match‐related injury rates tended to be highest for DFs and MFs, while career‐altering effects appeared most pronounced in FWs, with an exploratory, non‐significant tendency also for GKs. Injury rate and consequence should be considered separately. Although non‐contact injuries are most common overall, direct contact injuries were relatively frequent among professional FWs and DFs. Prevention and rehabilitation strategies could be tailored to each position.

**Level of Evidence:**

Level II, prospective cohort study.

**Abbreviations**
ACL: anterior cruciate ligament
ANOVA: analysis of variance
CI: confidence interval
DF: defender
FW: forward
GK: goalkeeper
IQR: interquartile range
IRR: incidence rate ratio
MF: midfielder
MI: multivariate imputation

## INTRODUCTION

Anterior cruciate ligament (ACL) injury is one of the most serious injuries in football, placing a heavy burden on players through long‐term absence from competition [26, 28], high medical costs [14, 43], increased risk of re‐injury [55, 56] and early onset of post‐traumatic osteoarthritis [12, 54]. Despite successful surgical reconstruction and rehabilitation, many athletes fail to return to their pre‐injury performance levels, making ACL injury a potentially career‐changing or career‐ending event [34, 52].

Previous studies of ACL injuries in football have traditionally focused on professional players, particularly those at the elite level [5, 17, 20, 21, 26, 39, 40, 46, 52]. However, using data collected in a nationwide ACL registry in Germany, our research group has demonstrated that both the risk of injury and the subsequent prognosis differ significantly across playing levels, including professional, semi‐professional and amateur athletes. Specifically, compared with professional and semi‐professional players, amateur players showed a higher incidence of ACL injuries and the lowest rate of return to their pre‐injury performance level [48, 51]. Despite these findings, playing position has not yet been sufficiently analysed. Football is a sport in which the physical demands, movement patterns and mechanical stress vary greatly across positions such as goalkeeper (GK), defender (DF), midfielder (MF) and forward (FW) [2, 5]. Although several studies have reported position‐specific injury incidence rates in professional leagues [20, 39, 40, 46], no previous studies have comprehensively investigated how characteristics specific to each position affect players' careers, the actual injury risk adjusted for exposure time and injury mechanisms and actions.

Therefore, the primary purpose of this study was to comprehensively describe the characteristics of ACL injuries by playing position in German football, using a large‐scale national registry. Specifically, we aimed to investigate differences in career impact among professional players using detailed long‐term performance data, match and training incidence rates in professional leagues and injury mechanisms and actions across all competition levels.

We hypothesized that the career impact would be serious for GKs due to the specific nature of their position and for FWs due to their physical demands. Considering the incidence rate adjusted for exposure time, DFs and MFs would be shown to be at the highest risk of ACL injury. In addition, injury mechanisms would also vary by position and competition level.

## METHODS

### Study design and data source

This study is a retrospective cohort study based on prospectively collected data from the nationwide ACL Registry in German Football (‘Kreuzbandregister im Deutschen Fußball’).

Injury data have been recorded in the ACL registry using a standardized method since the 2014–2015 season [50]. All male football players with a new ACL injury who had actively played in the German professional leagues (first to third men's professional league), in the Bavarian semi‐professional leagues (fourth to sixth men's semi‐professional league) or in the Bavarian amateur leagues (seventh men's amateur league) were included in the study. The study design was approved by the Ethics Committee of the University of Regensburg (ID: 22‐2807‐101), and informed consent was obtained from all participants.

### Data collection

As previously described [50], annually, football officials, clubs and team physicians were informed and invited to participate in the ACL registry in German Football. Players received a standardized questionnaire to collect detailed information on demographics, injury history and the specific circumstances of the current injury (e.g., mechanism, action) and report new ACL injuries to our study office. In parallel, the study team obtained a double‐check of the registration data from national media data using a standardized data collection method to minimize typical data loss and reduce the dropout rate [25, 26, 50]. Based on ACL registration data after each season, we analysed official match statistics to investigate the characteristics of injured players' return to competition. This allowed us to identify the timing of return to competition from official match records and compare it with the registered injury date. Post‐injury career data, including the number of matches and minutes played per season, were collected retrospectively for the pre‐injury season and prospectively for the subsequent three seasons. In this way, a record of match performance over five seasons was documented. Career outcomes such as a drop in competitive level, re‐injury and retirement within three seasons after injury were also recorded, although due to insufficient data for amateur players, the analysis of career data after injury focused solely on professional players (first to third leagues). The injury reports for this study followed the commonly used injury report protocol in football established by Fuller et al. [18] and were consistent with previous epidemiological injury studies of this study group [27, 30, 45].

### Participants

This study included male football players from the ACL registry between the 2016–2017 and 2021–2022 seasons who had sustained an acute ACL rupture while actively playing in Germany and for whom pre‐injury playing position was available. Players were classified into three competition levels based on their league at the time of injury, consistent with previous reports from this registry: professional leagues (first to third league), semi‐professional leagues (fourth to sixth league) and amateur leagues (seventh league) [48, 50]. For the primary analysis of this study, players were categorized into four groups based on their main playing position before injury: GK, DF, MF and FW.

### Incidence rate calculation

The injury incidence rate was calculated per 1000 player‐hours for both matches and training.

- Match exposure: Match exposure for professional players (first to third leagues) was calculated based on official match schedules for each season, as previously detailed [48].
- Training exposure: As individual training data were not available, training exposure was calculated based on previously published data from the same leagues and competition levels [24, 25, 27, 29, 30]. This approach provided a standardized estimate of exposure across seasons and leagues.
- Positional exposure: Because position‐specific exposure data were not available, the total league‐wide exposure hours for matches and training were allocated to each of the four playing positions based on the positional distribution of all registered players in each league for every season.

### Career outcome

Career outcomes were evaluated only among professional players and were assessed within three consecutive seasons following ACL injury. A drop in competitive level was defined as an individual player's movement to a lower league level. ACL re‐injury was defined as a subsequent ACL injury occurring in either the ipsilateral or contralateral knee. Retirement was defined as discontinuation of participation at the professional or semi‐professional level.

### Injury mechanism

Injury mechanism was categorized as previously described [49]. Direct contact injuries were defined as injuries resulting from a direct application of force from a teammate, opponent or object to the injured or adjacent region. Indirect contact injuries were defined as injuries in which an external force was applied by another player or object immediately before or during the injury and influenced the athlete's movement, thereby indirectly contributing to the injury. Non‐contact injuries were defined as injuries occurring without any direct or indirect external force from another player or object.

### Statistical analysis

All statistical analyses were performed using R statistical software via the EZR interface (Saitama Medical Center, Jichi Medical University) [23]. A *p* value of <0.05 was considered statistically significant.

Continuous variables were presented as mean ± standard deviation, except for the time to return to competition, which is presented as median (interquartile range [IQR]) due to outliers. The normality of data distribution was assessed using the Shapiro–Wilk test. For comparisons across the four position groups, one‐way analysis of variance (ANOVA) with Tukey's post hoc test was used for normally distributed data, while the Kruskal–Wallis test with Steel–Dwass post hoc test was used for non‐normally distributed data. However, for age at injury and anthropometric variables, ANOVA was used to compare groups to facilitate clearer interpretation, as this test is robust under the central limit theorem. Categorical variables were presented as counts and percentages. Differences in proportions between position groups were analysed using the chi‐squared test or Fisher's exact test, as appropriate, with Holm's adjustment for post hoc pairwise comparisons.

Variables with substantial missing data, specifically those on injury circumstances (e.g., mechanism, action), were analysed using 100 datasets imputed by multivariate imputation (MI) by chained equations. Associations were assessed using multinomial logistic regression, and results were combined using Rubin's rules. For all other variables, complete case analysis was performed.

To analyse the longitudinal data on matches and minutes played, linear mixed‐effects models were used, with position, time and their interaction as fixed effects, and a random intercept for each player. Incidence rates were compared between positions using Poisson regression, and incidence rate ratios (IRRs) with 95% confidence intervals were calculated. No formal sample size or power analysis was performed, as this study aimed to analyse all available cases within the registry to maximize statistical power. However, due to the limited number of GKs, analyses involving GKs were considered exploratory.

## RESULTS

### Participant characteristics

A total of 742 ACL‐injured players with available positional data were included in the study. Of these, 172 (23.2%) were classified as professional, 316 (42.6%) as semi‐professional and 254 (34.2%) as amateur. When stratified by playing position, the baseline characteristics at the time of injury differed significantly (Table 1). GKs were significantly older at the time of injury compared to FWs (mean 25.8 vs. 23.8 years, *p* = 0.015) and were also significantly taller and heavier than all other field players (*p* < 0.001).

**Table 1:** Baseline characteristics by playing position.

	GK (*n* = 34)	DF (*n* = 237)	MF (*n* = 294)	FW (*n* = 177)	*p* Value
Age (years)	25.81 ± 5.19	24.34 ± 3.81	23.69 ± 4.06	23.76 ± 3.72	0.015*
Height (cm)	189.24 ± 5.88	183.05 ± 6.38	179.29 ± 5.19	181.58 ± 5.84	<0.001*
Body weight (kg)	85.64 ± 7.97	79.01 ± 7.16	75.92 ± 6.43	77.62 ± 6.99	<0.001*
BMI (kg/m^2^)	23.96 ± 1.23	23.49 ± 1.73	23.55 ± 1.53	23.53 ± 1.63	0.786

*Note*: Values are presented as mean ± standard deviation. *p* Values were calculated using one‐way ANOVA.

Abbreviations: ANOVA, analysis of variance; BMI, body mass index; DF, defender; FW, forward; GK, goalkeeper; MF, midfielder.

* *p* Value < 0.05.

### Career impact and return to performance in professional players

As in the overall cohort, baseline characteristics at the time of injury varied significantly by position within the cohort of 172 professional players (Table 2).

**Table 2:** Baseline characteristics by playing position in professional players.

	GK (*n* = 11)	DF (*n* = 66)	MF (*n* = 55)	FW (*n* = 40)	*p* Value
Age (years)	26.67 ± 7.48	24.35 ± 3.60	25.33 ± 3.99	22.82 ± 3.87	0.012*
Height (cm)	188.73 ± 5.66	183.79 ± 6.59	179.47 ± 5.64	182.60 ± 5.78	<0.001*
Body weight (kg)	84.14 ± 7.56	79.26 ± 6.44	75.76 ± 6.74	78.32 ± 5.69	0.008*
BMI (kg/m^2^)	23.89 ± 1.28	23.34 ± 1.26	23.53 ± 1.09	23.29 ± 1.67	0.671

*Note*: Values are presented as mean ± standard deviation. *p* Values were calculated using one‐way ANOVA.

Abbreviations: ANOVA, analysis of variance; BMI, body mass; DF, defender; FW, forward; GK, goalkeeper; MF, midfielder.

* *p* Value < 0.05.

Career outcomes within three seasons post‐injury differed by position. GKs experienced a drop in competitive level at a relatively higher rate than all field players; however, this difference was not statistically significant after Holm correction. (GK: 54.5%, DF: 24.2%, MF: 29.1%, FW: 47.5%; unadjusted *p* = 0.033; adjusted *p* = 0.186 for GK vs. DF) (Figure 1a). ACL re‐injury rates within three seasons in professionals were approximately 15% for all field players, with no re‐injuries recorded for GKs (Figure 1b). The retirement rate was similar across all positions (Figure 1c).

**Figure 1:**
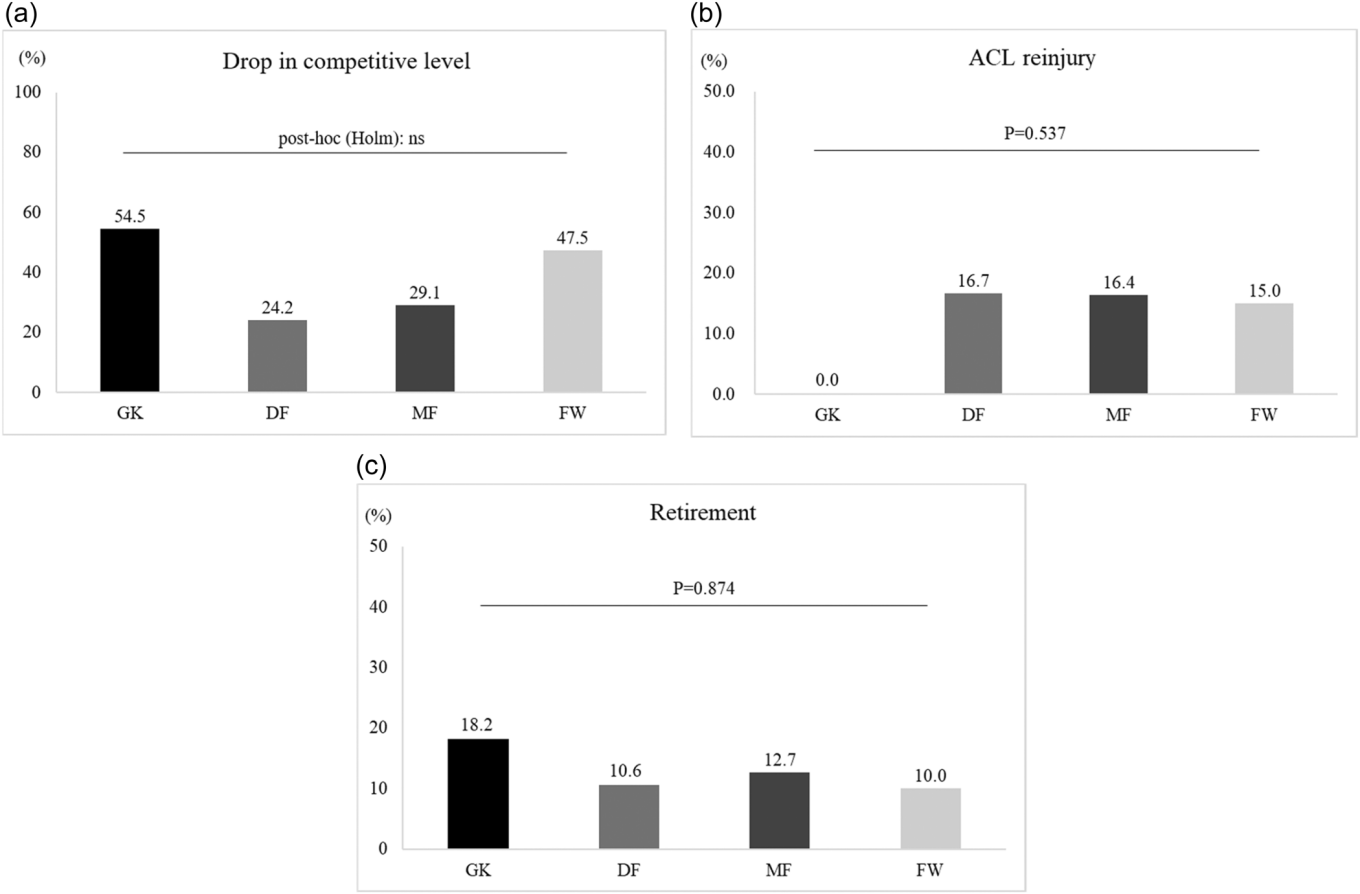
(a) Rates of a drop in competitive level within three seasons after ACL injury by position. This shows the proportion of players who experienced a drop in competitive level within three seasons after ACL injury across positions. Overall, GKs experienced a drop in competitive level at a relatively higher rate compared with field players, whereas DFs showed a relatively lower rate. The number of players in each position was GK (*n* = 11), DF (*n* = 66), MF (*n* = 55), FW (*n* = 40). Bars represent the percentage of players who were relegated to a lower league level. Percentages are calculated based on these denominators. The *p* value, obtained from a chi‐squared test, suggested a significant difference between positions (*p* = 0.033), but it was not significant after Holm correction. (b) ACL re‐injury rates within three seasons after ACL injury by position. This shows the proportion of players who experienced an ACL re‐injury within three seasons after ACL injury across positions. ACL re‐injury rates within three seasons were approximately 15% for all field players, with no re‐injuries recorded for GKs. The number of players in each position was GK (*n* = 11), DF (*n* = 66), MF (*n* = 55), FW (*n* = 40). Bars represent the percentage of players who sustained a subsequent ACL injury. Percentages are calculated based on these denominators. No significant difference was found in re‐injury rates between positions (chi‐squared test, *p* = 0.537). (c) Retirement rates within three seasons after ACL injury by position. This shows the proportion of players who retired from professional or semi‐professional football within three seasons after ACL injury across positions. The number of players in each position was GK (*n* = 11), DF (*n* = 66), MF (*n* = 55), FW (*n* = 40). Bars represent the percentage of players who retired from professional or semi‐professional football. Percentages are calculated based on these denominators. No significant differences in retirement rates were observed between positions (chi‐squared test, *p* = 0.874). ACL, anterior cruciate ligament; DF, defender; FW, forward; GK, goalkeeper; MF, midfielder; n.s., not significant.

Among professional players, the median time to return to competition was relatively longer for DFs (327 days) and shorter for MFs (286 days), although this difference was not statistically significant (*p* = 0.212) (Figure 2).

**Figure 2:**
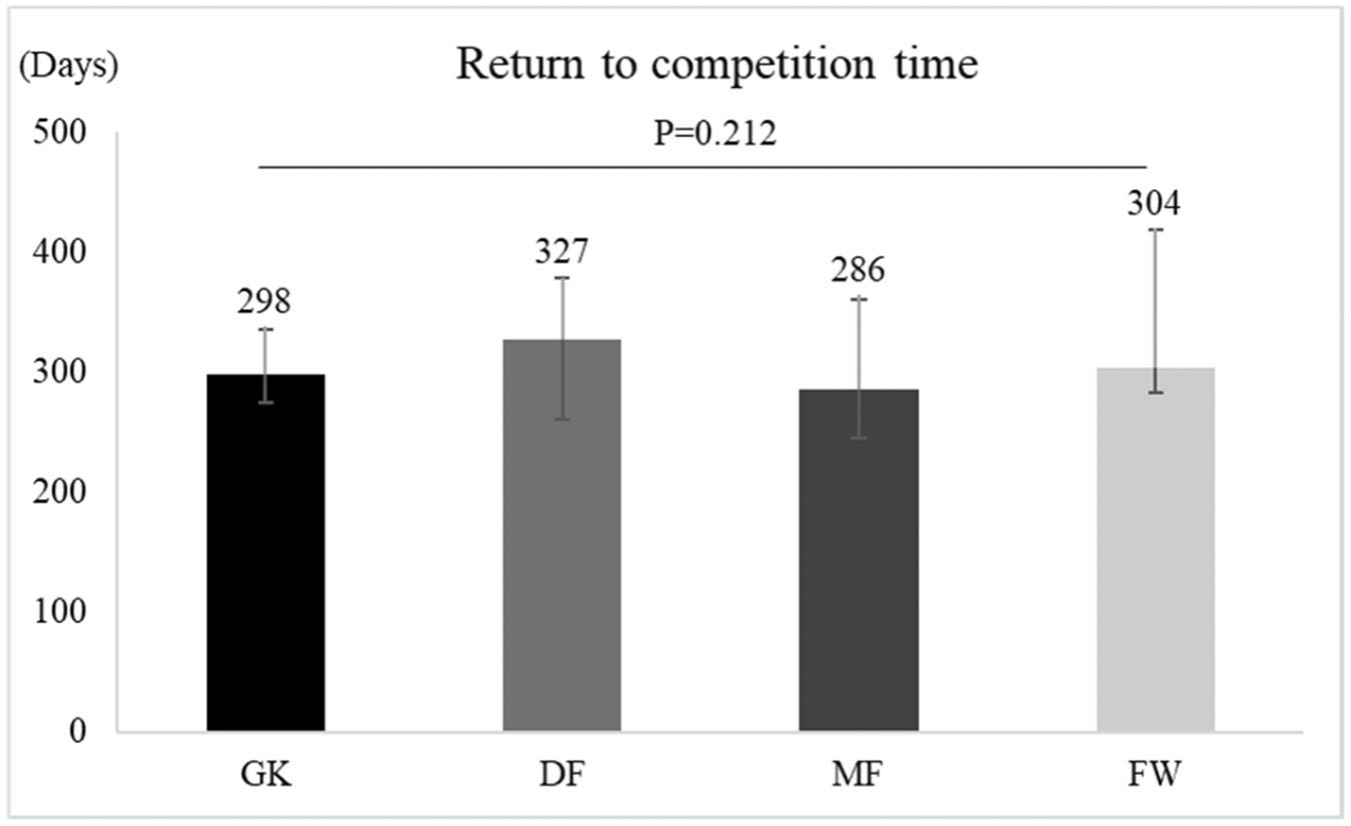
Time to return to competition by position after ACL injury. This shows time to return to competition after ACL injury across positions. The median time to return to competition was relatively longer for DFs and shorter for MFs. The number of players in each position was GK (*n* = 11), DF (*n* = 66), MF (*n* = 55), FW (*n* = 40). Bars represent the median time, and error bars indicate the interquartile range (IQR). *p* value was calculated using the Kruskal–Wallis test, showing no statistically significant difference between positions (*p* = 0.212). ACL, anterior cruciate ligament; DF, defender; FW, forward; GK, goalkeeper; MF, midfielder; n.s., not significant.

The longitudinal analysis of post‐injury performance suggested patterns based on position among professional players (Figure 3a,b). A linear mixed‐effects model revealed significant main effects for both time (*p* < 0.001) and position (*p* < 0.001), but the interaction between time and position was not statistically significant (*p* = 0.064 for matches played; *p* = 0.105 for minutes played) (see Supporting Information S1: Tables 1 and 2). Exploratory post hoc analysis may suggest that GKs' number of matches played tended to remain lower than that of all field players during the first two seasons after return (see Supplementary Tables S3–S5). While DFs and FWs appeared to regain their pre‐injury match counts by the second season, GKs tended to remain below even by the third season (see Supporting Information S1: Tables 4 and 5). A similar trend was observed for playing minutes, although these findings should be interpreted with caution due to the lack of a significant interaction (see Supporting Information S1: Tables 6–8).

**Figure 3:**
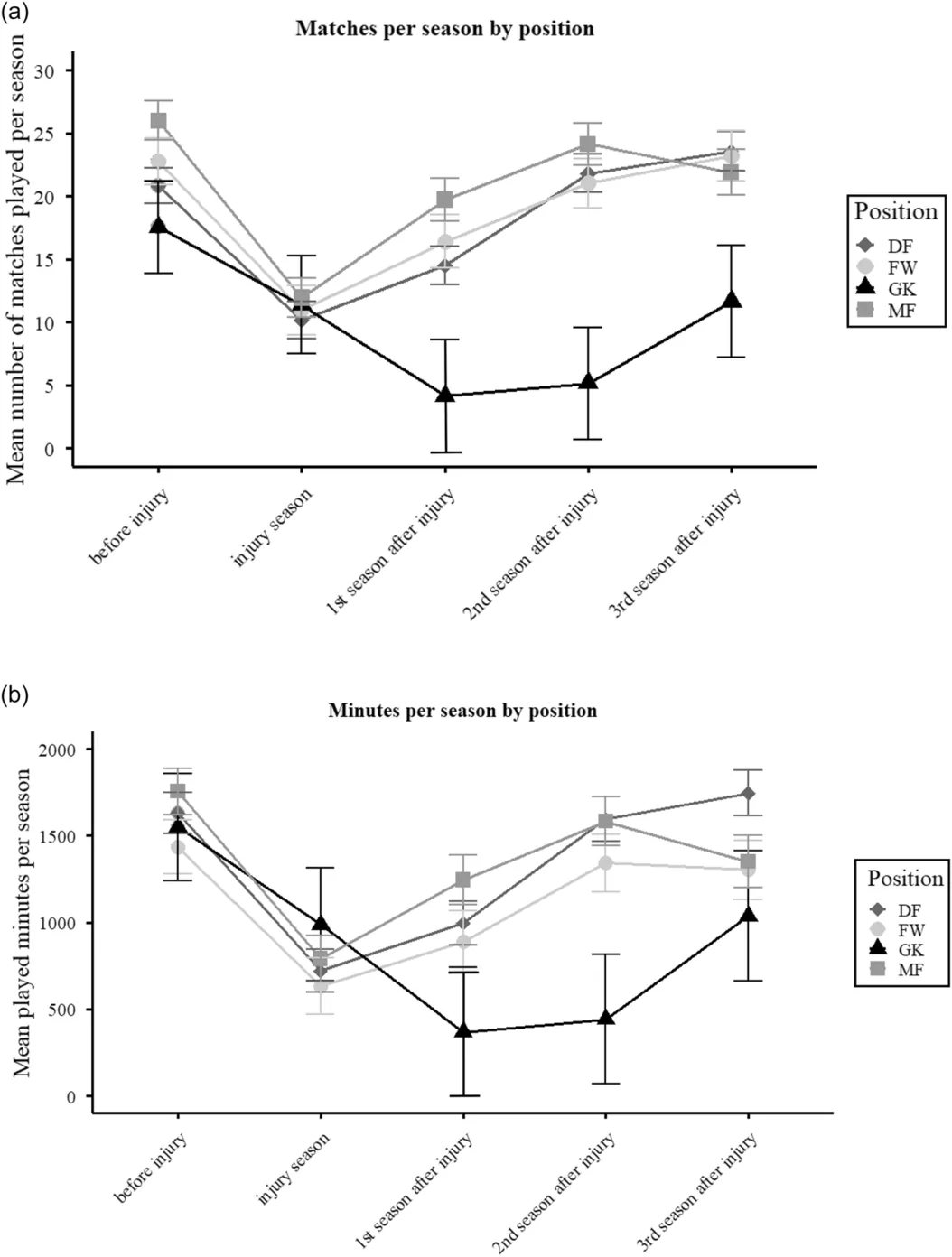
(a) Mean number of matches played per season by position. This shows longitudinal changes in the number of matches played per season across positions based on a linear mixed‐effects model. Estimates and 95% confidence intervals are shown for the season before injury, the injury season and three consecutive seasons after injury. The model showed significant main effects of both time (*p* < 0.001) and position (*p* < 0.001), while the interaction between time and position was not statistically significant (*p* = 0.064). Although GK estimates appeared descriptively lower than those of field players, this pattern was not supported by the statistical interaction. (b) Mean played minutes per season by position. This shows longitudinal changes in played minutes per season across positions based on a linear mixed‐effects model. Estimates and 95% confidence intervals are shown for the season before injury, the injury season and three consecutive seasons after injury. The model showed significant main effects of both time (*p* < 0.001) and position (*p* < 0.001), while the interaction between time and position was not statistically significant (*p* = 0.105). Although GK estimates appeared descriptively lower than those of field players, this pattern was not supported by the statistical interaction. DF, defender; FW, forward; GK, goalkeeper; MF, midfielder.

### Injury incidence in professional leagues

The incidence rates for ACL injury in professional leagues are shown in Tables 3 and 4. During matches, the highest incidence was among DFs (0.397 per 1000 player‐hours) and MFs (0.370), while the lowest was among GKs (0.167). Poisson regression analysis suggested that GKs (IRR = 0.422, 95% CI: 0.145–0.983, *p* = 0.071) and FWs (IRR = 0.577, 95% CI: 0.311–1.025, *p* = 0.068) experienced a lower incidence of match‐related ACL injuries, with the overall model showing a non‐significant trend (*p* = 0.081). Descriptive estimates suggested that training‐related incidence rates were similar across positions (*p* = 0.831) (see Supporting Information S1: Tables 9 and 10).

**Table 3:** Incidence and IRRs of match‐related ACL injuries.

Position	Estimated match incidence (per 1000 h)	IRR (vs. DF)	95% CI	*p* Value
DF	0.397	1.00 (Reference)	—	—
FW	0.229	0.577	(0.311, 1.025)	0.068
GK	0.167	0.422	(0.145, 0.983)	0.071
MF	0.370	0.932	(0.569, 1.517)	0.777

*Note*: IRRs were estimated by Poisson regression model, using DF as the reference group. Overall *p* value for the model = 0.081.

Abbreviations: ACL, anterior cruciate ligament; CIs, confidence intervals; DF, defender; FW, forward; GK, goalkeeper; IRRs, incidence rate ratios; MF, midfielder.

**Table 4:** Incidence and IRRs of training‐related ACL injuries.

Position	Estimated training incidence (per 1000 h)	IRR (vs. DF)	95% CI	*p* Value
DF	0.022	1.00 (Reference)	—	—
FW	0.023	1.033	(0.548, 1.923)	0.919
GK	0.018	0.806	(0.297, 1.868)	0.639
MF	0.017	0.790	(0.408, 1.497)	0.474

*Note*: IRRs were estimated by Poisson regression model, using DF as the reference group. Overall *p* value for the model = 0.831.

Abbreviations: ACL, anterior cruciate ligament; CIs, confidence intervals; DF, defender; FW, forward; GK, goalkeeper; IRRs, incidence rate ratios; MF, midfielder.

### Injury circumstances and competition level

Of the 742 recorded injuries, the injury circumstances data (game or training) were available for 488 cases. Among these, 379 (77.7%) occurred during games (see Supporting Information S1: Figure 1). Subsequent analyses of injury mechanisms and actions were therefore restricted to game‐related injuries due to the limited number of training injuries and the limited detail available for them. The extent of missing data for key variables is presented in Table 5.

**Table 5:** Summary of missing data for injury circumstances, mechanisms and actions.

Variables	Total	Missing *n* (%)
Injury circumstances (game/training)	742	254 (34.2)
Injury mechanisms (game only)	379	105 (27.7)
Injury actions (game only)	379	136 (35.9)

Regarding injury mechanisms, non‐contact injuries were the most common across all positions (*p* = 0.321, Figure 4a, see Supporting Information S1: Table 11). Among professional players, direct contact injuries appeared to be the largest single injury mechanism category for both DFs (46.2%) and FWs (66.7%) (*p* = 0.430, Figure 4b, see Supporting Information S1: Table 11). In contrast, non‐contact injuries were the most frequent mechanism across all positions in amateur players (*p* = 0.395, Figure 4d, see Supporting Information S1: Table 11). Among semi‐professional players, MFs showed a higher proportion of direct injuries than FWs (Holm‐adjusted *p* = 0.046, Figure 4c, see Supporting Information S1: Table 11). Analysis using MI was performed to estimate values after imputing missing data. Overall trends were consistent between the raw and MI datasets (see Supporting Information S1: Table 12 and Figure 2).

**Figure 4:**
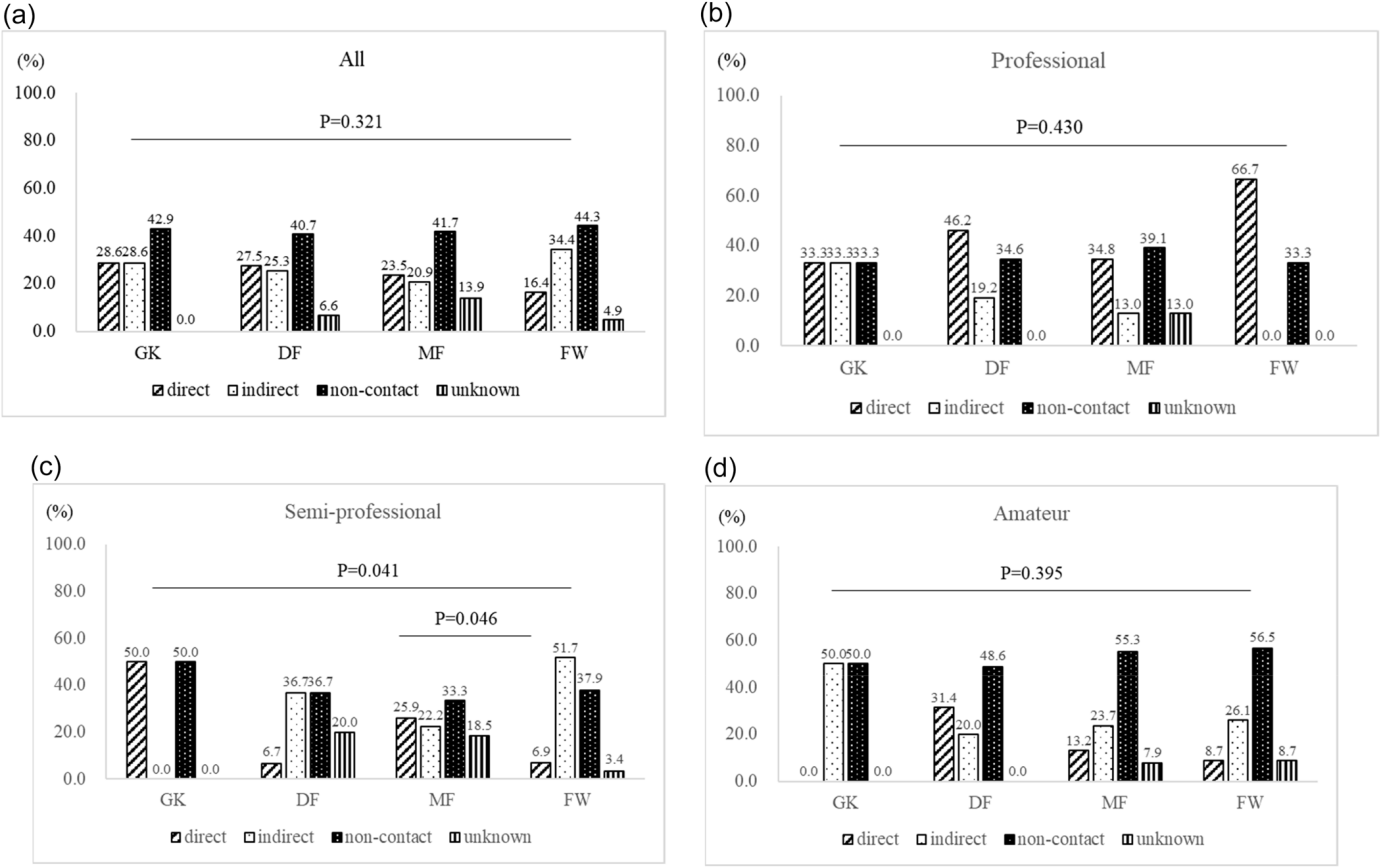
Distribution of ACL injury mechanisms during matches by position. (a) This shows the distribution of ACL injury mechanisms during matches across positions. Non‐contact injuries were the most frequent mechanism across all positions. The number of players in each position was GK (*n* = 7), DF (*n* = 91), MF (*n* = 115), FW (*n* = 61). Percentages for injury mechanisms are calculated based on these denominators. No significant differences were observed between positions (*p* = 0.321). (b) This shows the distribution of ACL injury mechanisms during matches across positions in professional players. Direct contact injuries were the most frequent injury mechanism among DFs and FWs. The number of players in each position was GK (*n* = 3), DF (*n* = 26), MF (*n* = 23), FW (*n* = 9). Percentages for injury mechanisms are calculated based on these denominators. No significant differences were observed between positions (*p* = 0.430). (c) This shows the distribution of ACL injury mechanisms during matches across positions in semi‐professional players. MFs had a higher proportion of direct injuries than FW (*p* = 0.046, Holm‐adjusted). The number of players in each position was GK (*n* = 2), DF (*n* = 30), MF (*n* = 54), FW (*n* = 29). Percentages for injury mechanisms are calculated based on these denominators. (d) This shows the distribution of ACL injury mechanisms during matches across positions in amateur players. Non‐contact injuries were the most frequent mechanism across all positions. The number of players in each position was GK (*n* = 2), DF (*n* = 35), MF (*n* = 38), FW (*n* = 23). Percentages for injury mechanisms are calculated based on these denominators. No significant differences were observed between positions (*p* = 0.395). ACL, anterior cruciate ligament; DF, defender; FW, forward; GK, goalkeeper; MF, midfielder.

Regarding injury‐related actions, tackling was the most common action leading to ACL injury overall. However, position‐specific actions were observed descriptively. For DFs, tackling and heading were common, while for FWs, dribbling and shooting were common, and for MFs, dribbling and ball reception were common. At the same time, a substantial proportion of actions were classified as Other, which could not be assigned to predefined categories, particularly in GKs. These differences were not statistically significant after Holm correction (Figure 5, Supporting Information S1: Table 13). Overall trends were consistent between the raw and MI datasets (see Supporting Information S1: Table 14 and Figure 3).

**Figure 5:**
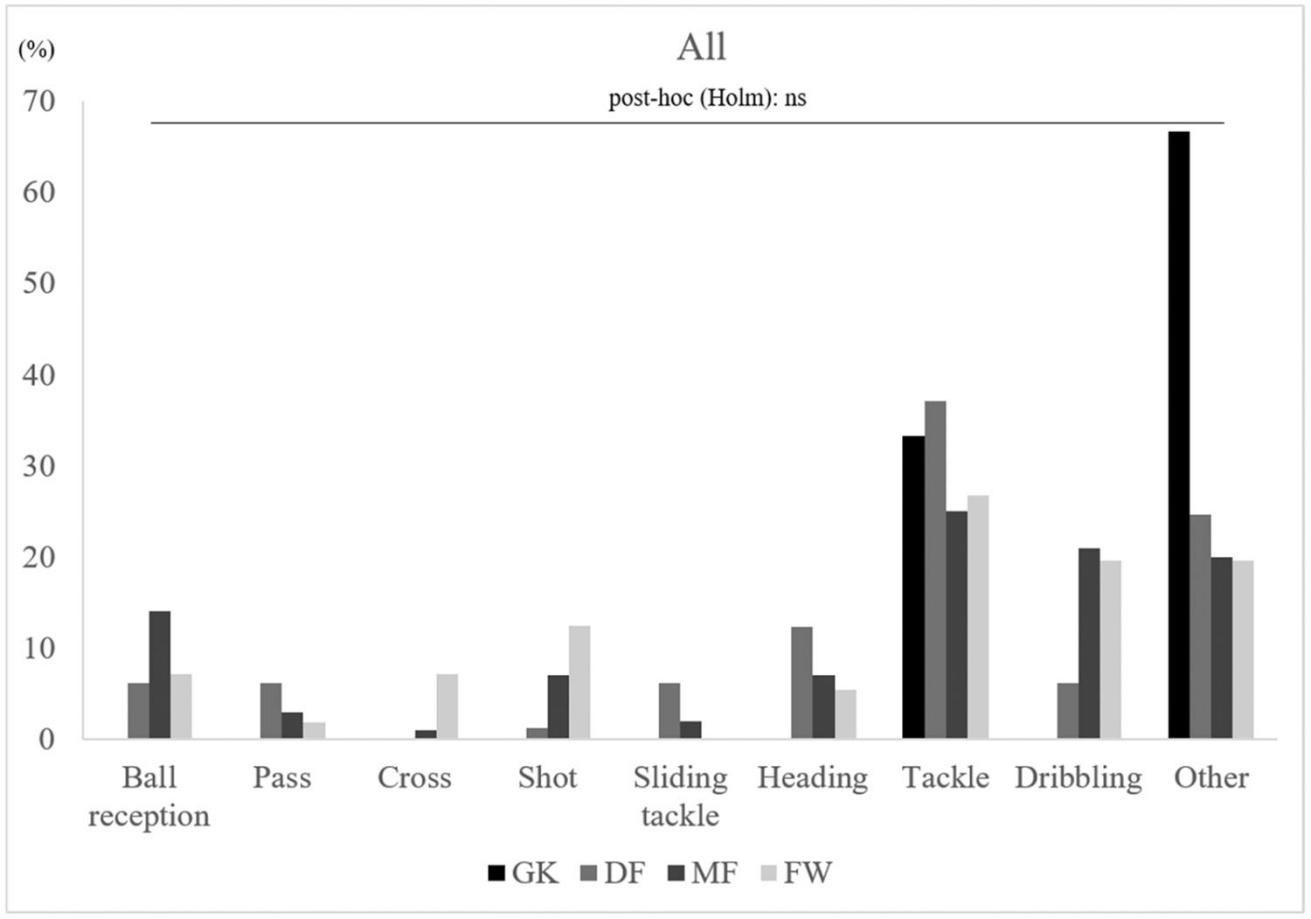
Distribution of ACL injury‐related actions by position. This shows the distribution of ACL injury‐related actions across positions. Overall, tackling was the most common action leading to ACL injury, although the distribution of actions varied by position. A substantial proportion of actions were classified as Other, which could not be assigned to predefined categories, particularly in GKs. The number of players in each position was GK (*n* = 6), DF (*n* = 81), MF (*n* = 100), FW (*n* = 56). Percentages for injury actions are calculated based on these denominators. Although overall differences were significant (chi‐squared test, *p* = 0.002), no pairwise comparisons remained significant after Holm correction. ACL, anterior cruciate ligament; DF, defender; FW, forward; GK, goalkeeper; MF, midfielder.

## DISCUSSION

This study, based on a large national ACL registry, is the first to comprehensively describe the characteristics and outcomes of ACL injuries by position across all levels of German football. We identified several specific patterns in these outcomes according to position and competition level. The three main findings were: (1) a potentially greater career impact in professional GKs and FWs, although the results concerning GKs should be interpreted with caution due to the limited sample size. (2) higher observed proportions of match‐related injuries in professional DFs and MFs. (3) higher observed proportions of direct contact injuries in professional FWs and DFs compared with semi‐professional and amateur levels.

Among professional players, the major consequence of ACL injuries for career progression varies according to the position. Previous reports have indicated that GKs have a lower ACL injury rate and lower risk compared to other positions [20, 40, 46]. Our study also observed a lower incidence rate among GKs. At the same time, a 54.5% rate of drop in competitive level and a similar pattern in match participation were observed during the seasons after injury. These observations may hypothetically suggest that, in the unique position of GK, long‐term absences could contribute to loss of the starting GK position; however, given the very small number of GKs, this interpretation remains speculative. While previous research has reported that GKs have lower retirement rates than FWs, our findings suggest a potentially different career impact of ACL injuries on GKs [6]. Unlike field players who are frequently substituted, teams usually rely on a single starting GK. Long rehabilitation periods might allow substitute players to secure the starting GK position, which could make return more difficult for injured GKs, although this proposed mechanism is exploratory and not statistically supported. In addition, GKs tend to be taller and heavier, and more prone to injury at a relatively older age, which may be associated with return to competition outcomes [4]. Due to the limited number of GKs in our study, many statistical comparisons did not reach significance. Therefore, analyses involving GKs should be considered exploratory and interpreted with caution. Nevertheless, the observed trends were generally consistent across multiple outcomes.

FWs also face an increased risk of career slowdown, as indicated by a significantly younger age at injury. A relatively higher rate of drop in competitive level (47.5%) was also observed. FWs are reported to have more high‐intensity stops, sprints and changes of direction than other positions, and also experience more physical contact [5, 11, 19, 41]. These physical characteristics are believed to be slightly impaired after ACL reconstruction [3]. As a result, playing time may not return to pre‐injury levels even after 2–3 seasons of recovery. Several previous studies also suggest a long‐term decline in performance for FWs after returning from ACL injury [13, 16].

In contrast to GKs and FWs, DFs demonstrated a relatively high career stability and a low rate of drop in competitive level among all positions and reportedly recovered most smoothly after ACL surgery [16]. Our study also showed that DFs had a relatively high incidence of ACL injuries related to matches, consistent with previous studies [20, 35, 39, 40]. This may be due to the important skills needed to play in defence. Compared to other positions, DFs spend the most time moving backward and laterally, change direction the most and experience the most rotational stress on their knees [5]. Furthermore, regardless of position, injuries often occur during defensive plays, and the injury mechanism is frequently a tackle [10, 36, 42, 57]. These findings indicate that the main tasks of DFs can be a direct risk factor for ACL injury, resulting in a high injury rate. On the other hand, while agility is important for DFs, skills such as positioning, understanding tactics, physical fitness and jumping ability are equally important. Because these characteristics are less affected by the subtle neuromuscular dysfunction that may remain after ACL surgery, DFs might be able to adapt their playing style more effectively and maintain their performance level.

Furthermore, MFs had a relatively high incidence of ACL injuries during matches, consistent with previous reports [20, 39, 40, 46]. In the case of MFs, this may be due to their role as the team's playmaker. They run the longest distances, perform many sprints and large changes of direction, and also perform many risky pivot movements, participating in both attack and defence [5, 11, 19, 42]. As our results show, this high‐intensity play and constant involvement in both attack and defence may increase the risk of fatigue‐related non‐contact injuries, thereby contributing to the high injury rate [8, 9]. However, in terms of prognosis, the recovery period was short, the number of matches and playing time recovered quickly and the rate of drop in competitive level was relatively low. Reportedly, their performance may even improve over the 3–4 seasons after an injury [16], and like DFs, MFs are also believed to adapt their playing style and maintain their performance level.

Another new finding in this study is that the mechanisms of injury differ depending on the position and level of competition. A relatively high number of direct‐contact injuries among professional DFs and FWs suggests the physical intensity and aggressive nature of tackles and duels at the elite level [42]. This may indicate that, for professional DFs and FWs, prevention strategies need to address not only neuromuscular control during non‐contact situations but also the physical stress during player‐to‐player contact situations [3, 7]. Specifically, preventative programmes aimed at maintaining knee alignment during player‐to‐player contact, such as core stability training, perturbation‐based balance exercises and position‐specific contact drills, could be beneficial [1, 15, 22]. For MFs who exhibited the highest proportion of non‐contact injuries across level of competition, fatigue‐resistant neuromuscular training including repeated cutting, deceleration and landing tasks may be beneficial [58, 59]. For GKs, although the detailed data were limited, training focusing on landing mechanics and change‐of‐direction control could also be considered. Some reports indicate that appropriate prevention training programmes significantly reduced severe knee injuries in elite football, but others argue that the transition from theoretical knowledge to practical routines is not optimal, suggesting the importance of position‐specific prevention [25, 29, 31, 47]. Recent editorials in this journal have similarly emphasized that the principal challenge in ACL prevention lies less in the strength of the evidence than in closing the implementation gap between proven programmes and everyday practice and have called for wide‐scale adoption of the ESSKA‐ESMA ‘Prevention for All’ ACL programme, including in youth and lower‐level football [37, 38]. On the other hand, the high number of non‐contact injuries in all positions among amateur players suggests another underlying problem. A study including many amateur football players found that most injuries were caused by non‐contact mechanisms such as pivoting and landing [42]. This suggests that physical conditioning and neuromuscular control are generally lower at the semi‐professional and amateur levels than among professional players, and that knee injuries therefore often occur during players' own cutting and landing movements before contact with an opponent [42, 49]. This emphasizes the urgent need to use structured, evidence‐based neuromuscular rehabilitation programmes more widely at the semi‐professional and amateur levels [32, 33, 60]. Despite the high percentage of missing data, the results of MI analysis suggest these patterns and indicate the need for prevention measures suitable for each level, although some residual bias may remain.

Our findings regarding performance recovery are consistent with previous studies, indicating that return to competition does not necessarily mean returning to pre‐injury performance levels [34, 44, 52]. In addition, by analysing results by position, our study provides a more detailed understanding of career trajectories after injury. Similarly, while the incidence and mechanism data in professional football are comparable to those reported in other elite populations [20, 36, 39, 40, 42, 52, 53], our findings suggest differences in risk depending on playing level and position.

The strengths of this study include its large sample size, its coverage of all competitive levels from amateur to professional and its comprehensive analysis combining detailed injury data with objective performance measures. The use of a national registry, validated dual‐source data collection methods and MI for missing data improves the reliability of our findings.

Several limitations should be acknowledged. First, training exposure was estimated based on previously published data from the same leagues, as position‐specific exposure data were not available. Therefore, the use of estimated exposure may introduce potential bias, particularly if training loads differ across positions. Second, findings related to GKs should be interpreted with caution due to the limited number of injuries and reduced statistical power. Third, since this study is registry‐based, there was no non‐injured control group, making it impossible to determine risk ratios. Fourth, some data on specific injury circumstances were missing, particularly for training injuries and lower‐level players. Although MI was used in the exploratory analysis to address this issue, the relatively high proportion of missingness may have introduced some uncertainty in the estimates. Therefore, these results should be interpreted with caution. Fifth, information on concomitant injuries, graft type, surgical technique and rehabilitation protocols was not available, which may have influenced outcomes. Finally, this study included only male football players; therefore, the generalizability of the findings to female players is limited.

## CONCLUSION

This comprehensive analysis using nationwide ACL registration data showed that the characteristics and prognosis of ACL injuries in football may vary by position and level of competition. The career impact may be most severe for professional FWs, with exploratory findings also suggesting a possible impact in GKs, while DFs and MFs face the highest injury risk during matches. It is important to understand that injury incidence and injury consequences are different. Although most injuries occur through non‐contact mechanisms overall, a relatively high proportion of direct contact injuries was observed among professional FWs and DFs. These findings suggest that both injury prevention and post‐injury rehabilitation could be tailored according to a player's position and level in order to better protect their careers.

## AUTHOR CONTRIBUTIONS

**Shoichi Hasegawa**: Formal analysis; writing—original draft. **Werner Krutsch**: Methods; study design; conduction and supervision. **Hidejuki Koga**: Supervision; writing—review. **Volker Alt**: Supervision; methods; writing—revision. **Dominik Szymski**: Study design; conduction; analysis; writing—original draft. **Lorenz Huber**: Formal analysis; graphics; writing—original draft. All authors read and approved the final manuscript.

## CONFLICT OF INTEREST STATEMENT

The authors declare no conflict of interest.

## ACKNOWLEDGEMENTS

The authors received funding for this project from the Verwaltungs‐Berufsgenossenschaften (VBG).

## ETHICS STATEMENT

The study was approved by the Ethics Committee of the University of Regensburg (ID: 22‐2807‐101). All participants provided informed consent.

## Supporting information

Supporting file 1.

## Data Availability

Data are available on request.
