# Supplementary material for: Impact of playing position on ACL injury characteristics, risk and career outcomes in football—An analysis of 742 cases of the anterior cruciate ligament registry in German football
Source: Knee Surg Sports Traumatol Arthrosc. 2026 Aug 3;34(10):3794–806. doi: 10.1002/ksa.70566 (PMC13622896; doi:10.1002/ksa.70566)
Supplement: Supplementary file 1 [file KSA-34-3794-s001.docx]

**Supplementary Table & Figure**

**Supplementary Table S1**. **Type III Analysis of Variance for the Linear Mixed-Effects Model (Matches)**

| Effect | Num df | Den df | F value | P value |
| --- | --- | --- | --- | --- |
| Position | 3 | 148.4 | 6.04 | <0.001* |
| TimePoint | 4 | 527.8 | 15.98 | <0.001* |
| Position × TimePoint | 12 | 525.7 | 1.7 | 0.064 |

Effects of Position, Time point, and their interaction are shown with F values, degrees of freedom, and P values.

* P value < 0.05

**Supplementary Table S2**. **Type III Analysis of Variance for the Linear Mixed-Effects Model (Minutes)**

| Effect | Num df | Den df | F value | P value |
| --- | --- | --- | --- | --- |
| Position | 3 | 150.9 | 2.5 | 0.062 |
| Time Point | 4 | 528.1 | 13.55 | <0.001* |
| Position × Time Point | 12 | 525.8 | 1.54 | 0.105 |

Effects of Position, Time point, and their interaction are shown with F values, degrees of freedom, and P values.

* P value < 0.05

**Supplementary Table S3. Linear Mixed-Effects Model Estimates for Matches Played per Season**

| Predictor | Estimate (95% CI) | P value |
| --- | --- | --- |
| Time Point (ref: Pre) |  |  |
| Injury | -10.65 (-14.27, -7.04) | <0.001* |
| 1st season | -6.30 (-9.96, -2.63) | <0.001* |
| 2nd season | 1.01 (-2.68, 4.70) | 0.593 |
| 3rd season | 2.74 (-1.01, 6.50) | 0.153 |
| Position (ref: DF) |  |  |
| FW vs DF | 1.95 (-2.57, 6.47) | 0.398 |
| GK vs DF | -3.25 (-10.93, 4.44) | 0.408 |
| MF vs DF | 5.20 (1.08, 9.33) | 0.014* |
| Time Point × Position interaction |  |  |
| FW × Injury | -1.16 (-7.12, 4.79) | 0.702 |
| GK × Injury | 4.46 (-5.69, 14.61) | 0.389 |
| MF × Injury | -3.42 (-8.76, 1.92) | 0.210 |
| FW × 1st | -0.04 (-6.25, 6.16) | 0.990 |
| GK × 1st | -7.11 (-18.14, 3.92) | 0.207 |
| MF × 1st | 0.02 (-5.50, 5.55) | 0.993 |
| FW × 2nd | -2.72 (-8.76, 3.32) | 0.377 |
| GK × 2nd | -13.41 (-24.45, -2.38) | 0.018* |
| MF × 2nd | -2.87 (-8.37, 2.63) | 0.307 |
| FW × 3rd | -2.29 (-8.45, 3.87) | 0.467 |
| GK × 3rd | -8.65 (-19.71, 2.41) | 0.126 |
| MF × 3rd | -6.85 (-12.53, -1.16) | 0.019* |

Estimates are shown as regression coefficients (β) with 95% CI. Pre and DF were used as reference categories for time point and position, respectively. Interaction terms show how changes differ from the reference group.

* P value < 0.05.

CI, confidence intervals; DF, defender; MF, midfielder; FW, forward; GK, goalkeeper.

**Supplementary Table S4. Pairwise Comparisons of Positions Within Each Time Point for Matches Played**

| Time Point | Comparison | Mean Difference | SE | P value |
| --- | --- | --- | --- | --- |
| Pre | DF vs FW | -1.95 | 2.31 | 0.833 |
|  | DF vs GK | 3.25 | 3.92 | 0.841 |
|  | DF vs MF | -5.20 | 2.11 | 0.066 |
|  | FW vs GK | 5.19 | 4.09 | 0.583 |
|  | FW vs MF | -3.25 | 2.41 | 0.531 |
|  | GK vs MF | -8.45 | 3.98 | 0.147 |
| Injury | DF vs FW | -0.79 | 2.42 | 0.988 |
|  | DF vs GK | -1.22 | 4.14 | 0.991 |
|  | DF vs MF | -1.78 | 2.14 | 0.839 |
|  | FW vs GK | -0.43 | 4.33 | 0.999 |
|  | FW vs MF | -1.00 | 2.49 | 0.978 |
|  | GK vs MF | -0.57 | 4.18 | 0.999 |
| 1st season | DF vs FW | -1.91 | 2.57 | 0.880 |
|  | DF vs GK | 10.36 | 4.69 | 0.122 |
|  | DF vs MF | -5.23 | 2.27 | 0.099 |
|  | FW vs GK | 12.27 | 4.91 | 0.061 |
|  | FW vs MF | -3.32 | 2.70 | 0.608 |
|  | GK vs MF | -15.58 | 4.76 | 0.006* |
| 2nd season | DF vs FW | 0.77 | 2.48 | 0.989 |
|  | DF vs GK | 16.66 | 4.70 | 0.002* |
|  | DF vs MF | -2.33 | 2.25 | 0.728 |
|  | FW vs GK | 15.89 | 4.86 | 0.006* |
|  | FW vs MF | -3.11 | 2.58 | 0.623 |
|  | GK vs MF | -18.99 | 4.75 | <0.001* |
| 3rd season | DF vs FW | 0.34 | 2.55 | 0.999 |
|  | DF vs GK | 11.90 | 4.71 | 0.057 |
|  | DF vs MF | 1.65 | 2.37 | 0.900 |
|  | FW vs GK | 11.56 | 4.89 | 0.085 |
|  | FW vs MF | 1.31 | 2.70 | 0.963 |
|  | GK vs MF | -10.25 | 4.79 | 0.142 |

Pairwise comparisons were based on estimated marginal means from the linear mixed-effects model.

P values were adjusted for multiple comparisons using the Tukey method.

SE standard error; DF, defender; MF, midfielder; FW, forward; GK, goalkeeper.

**Supplementary Table S5. Pairwise Comparisons of Time Points Within Each Position for Matches Played**

| Position | Comparison | Mean Difference | SE | P value |
| --- | --- | --- | --- | --- |
| DF | Pre vs Injury | 10.65 | 1.84 | <0.001* |
|  | Pre vs 1st | 6.30 | 1.87 | 0.007* |
|  | Pre vs 2nd | -1.01 | 1.88 | 0.984 |
|  | Pre vs 3rd | -2.74 | 1.92 | 0.608 |
|  | Injury vs 1st | -4.36 | 1.91 | 0.154 |
|  | Injury vs 2nd | -11.66 | 1.93 | <0.001* |
|  | Injury vs 3rd | -13.40 | 1.96 | <0.001* |
|  | 1st vs 2nd | -7.30 | 1.94 | 0.002* |
|  | 1st vs 3rd | -9.04 | 1.98 | <0.001* |
|  | 2nd vs 3rd | -1.74 | 1.98 | 0.906 |
| FW | Pre vs Injury | 11.81 | 2.42 | <0.001* |
|  | Pre vs 1st | 6.34 | 2.55 | 0.097 |
|  | Pre vs 2nd | 1.72 | 2.44 | 0.956 |
|  | Pre vs 3rd | -0.46 | 2.49 | 1.000 |
|  | Injury vs 1st | -5.48 | 2.62 | 0.227 |
|  | Injury vs 2nd | -10.10 | 2.52 | <0.001* |
|  | Injury vs 3rd | -12.27 | 2.57 | <0.001* |
|  | 1st vs 2nd | -4.62 | 2.64 | 0.404 |
|  | 1st vs 3rd | -6.79 | 2.68 | 0.085 |
|  | 2nd vs 3rd | -2.17 | 2.56 | 0.916 |
| GK | Pre vs Injury | 6.190 | 4.84 | 0.704 |
|  | Pre vs 1st | 13.41 | 5.31 | 0.086 |
|  | Pre vs 2nd | 12.41 | 5.31 | 0.135 |
|  | Pre vs 3rd | 5.91 | 5.31 | 0.800 |
|  | Injury vs 1st | 7.22 | 5.41 | 0.670 |
|  | Injury vs 2nd | 6.22 | 5.41 | 0.780 |
|  | Injury vs 3rd | -0.28 | 5.41 | 1.000 |
|  | 1st vs 2nd | -1.00 | 5.72 | 1.000 |
|  | 1st vs 3rd | -7.50 | 5.72 | 0.684 |
|  | 2nd vs 3rd | -6.50 | 5.72 | 0.787 |
| MF | Pre vs Injury | 14.07 | 2.00 | <0.001* |
|  | Pre vs 1st | 6.27 | 2.11 | 0.026* |
|  | Pre vs 2nd | 1.862 | 2.08 | 0.899 |
|  | Pre vs 3rd | 4,103 | 2.18 | 0.327 |
|  | Injury vs 1st | -7.80 | 2.11 | 0.002* |
|  | Injury vs 2nd | -12.21 | 2.08 | <0.001* |
|  | Injury vs 3rd | -9.97 | 2.18 | <0.001* |
|  | 1st vs 2nd | -4.41 | 2.17 | 0.253 |
|  | 1st vs 3rd | -2.17 | 2.26 | 0.874 |
|  | 2nd vs 3rd | 2.24 | 2.24 | 0.854 |

Pairwise comparisons were based on estimated marginal means from the linear mixed-effects model.

P values were adjusted for multiple comparisons using the Tukey method.

* P value < 0.05

SE standard error; DF, defender; MF, midfielder; FW, forward; GK, goalkeeper.

**Supplementary Table S6. Linear Mixed-Effects Model Estimates for Playing Minutes per Season**

| Predictor | Estimate (95% CI) | P value |
| --- | --- | --- |
| TimePoint (ref: Pre) |  |  |
| Injury | -913.32 (-1212.67, -613.97) | <0.001* |
| 1st season | -636.37 (-940.34, -332.39) | <0.001* |
| 2nd season | -36.53 (-342.29, 269.24) | 0.815 |
| 3rd season | 113.07 (-198.32, 424.47) | 0.477 |
| Position (ref: DF) |  |  |
| FW vs DF | -198.75 (-580.27, 182.77) | 0.308 |
| GK vs DF | -83.58 (-732.24, 565.09) | 0.801 |
| MF vs DF | 122.14 (-226.12, 470.40) | 0.492 |
| TimePoint × Position |  |  |
| FW × Injury | 109.32 (-384.04, 602.68) | 0.664 |
| GK × Injury | 351.09 (-490.02, 1192.20) | 0.414 |
| MF × Injury | -50.91 (-493.01, 391.19) | 0.822 |
| FW × 1st | 92.93 (-421.38, 607.24) | 0.723 |
| GK × 1st | -545.80 (-1460.75, 369.15) | 0.243 |
| MF × 1st | 127.13 (-330.85, 585.12) | 0.587 |
| FW × 2nd | -54.44 (-554.98, 446.11) | 0.831 |
| GK × 2nd | -1071.97 (-1987.52, -156.43) | 0.022* |
| MF × 2nd | -136.33 (-591.88, 319.21) | 0.558 |
| FW × 3rd | -243.13 (-753.61, 267.34) | 0.351 |

Estimates are shown as regression coefficients (β) with 95% CI. Pre and DF were used as reference categories for time point and position, respectively. Interaction terms show how changes differ from the reference group.

* P value < 0.05

CI, confidence intervals; DF, defender; MF, midfielder; FW, forward; GK, goalkeeper.

**Supplementary Table S7. Pairwise Comparisons of Positions Within Each Time Point for Playing Minutes**

| Time Point | Comparison | Mean Difference (min) | SE | P value |
| --- | --- | --- | --- | --- |
| Pre | DF vs FW | 198.8 | 195 | 0.737 |
|  | DF vs GK | 83.6 | 331 | 0.994 |
|  | DF vs MF | -122.1 | 178 | 0.902 |
|  | FW vs GK | -115.2 | 345 | 0.987 |
|  | FW vs MF | -320.9 | 203 | 0.392 |
|  | GK vs MF | -205.7 | 336 | 0.928 |
| Injury | DF vs FW | 89.4 | 204 | 0.972 |
|  | DF vs GK | -267.5 | 349 | 0.870 |
|  | DF vs MF | -71.2 | 181 | 0.979 |
|  | FW vs GK | -356.9 | 365 | 0.763 |
|  | FW vs MF | -160.7 | 210 | 0.870 |
|  | GK vs MF | 196.3 | 353 | 0.945 |
| 1st season | DF vs FW | 105.8 | 217 | 0.962 |
|  | DF vs GK | 629.4 | 395 | 0.383 |
|  | DF vs MF | -249.3 | 191 | 0.562 |
|  | FW vs GK | 523.6 | 414 | 0.585 |
|  | FW vs MF | -355.1 | 227 | 0.401 |
|  | GK vs MF | -878.7 | 401 | 0.127 |
| 2nd season | DF vs FW | 253.2 | 209 | 0.620 |
|  | DF vs GK | 1155.6 | 395 | 0.019* |
|  | DF vs MF | 14.2 | 190 | 1.000 |
|  | FW vs GK | 902.4 | 409 | 0.123 |
|  | FW vs MF | -239 | 217 | 0.689 |
|  | GK vs MF | -1141.4 | 400 | 0.023* |
| 3rd season | DF vs FW | 441.9 | 215 | 0.170 |
|  | DF vs GK | 708.7 | 397 | 0.281 |
|  | DF vs MF | 395.7 | 200 | 0.196 |
|  | FW vs GK | 266.8 | 411 | 0.916 |
|  | FW vs MF | -46.2 | 227 | 0.997 |
|  | GK vs MF | -313 | 403 | 0.865 |

Pairwise comparisons were based on estimated marginal means from the linear mixed-effects model.

P values were adjusted for multiple comparisons using the Tukey method.

* P value < 0.05

SE, standard error; DF, defender; MF, midfielder; FW, forward; GK, goalkeeper.

**Supplementary Table S8. Pairwise Comparisons of Time Points Within Each Position for Playing Minutes**

| Position | Comparison | Mean Difference (min) | SE | P value |
| --- | --- | --- | --- | --- |
| DF | Pre vs Injury | 913.3 | 153 | <0.001* |
|  | Pre vs 1st | 636.4 | 155 | <0.001* |
|  | Pre vs 2nd | 36.5 | 156 | 0.999 |
|  | Pre vs 3rd | -113.1 | 159 | 0.954 |
|  | Injury vs 1st | -277.0 | 159 | 0.406 |
|  | Injury vs 2nd | -876.8 | 160 | <0.001* |
|  | Injury vs 3rd | -1026.4 | 162 | <0.001* |
|  | 1st vs 2nd | -599.8 | 161 | 0.002* |
|  | 1st vs 3rd | -749.4 | 164 | <0.001* |
|  | 2nd vs 3rd | -149.6 | 164 | 0.893 |
| FW | Pre vs Injury | 804.0 | 200 | <0.001* |
|  | Pre vs 1st | 543.4 | 212 | 0.078 |
|  | Pre vs 2nd | 91.0 | 202 | 0.992 |
|  | Pre vs 3rd | 130.1 | 206 | 0.970 |
|  | Injury vs 1st | -260.6 | 218 | 0.753 |
|  | Injury vs 2nd | -713.0 | 209 | 0.006* |
|  | Injury vs 3rd | -673.9 | 213 | 0.014* |
|  | 1st vs 2nd | -452.5 | 219 | 0.236 |
|  | 1st vs 3rd | -413.4 | 222 | 0.341 |
|  | 2nd vs 3rd | 39.1 | 212 | 1.000 |
| GK | Pre vs Injury | 562.2 | 401 | 0.627 |
|  | Pre vs 1st | 1182.2 | 441 | 0.058 |
|  | Pre vs 2nd | 1108.5 | 441 | 0.088 |
|  | Pre vs 3rd | 512.0 | 441 | 0.773 |
|  | Injury vs 1st | 619.9 | 449 | 0.640 |
|  | Injury vs 2nd | 546.3 | 449 | 0.741 |
|  | Injury vs 3rd | -50.2 | 449 | 1.000 |
|  | 1st vs 2nd | -73.7 | 473 | 1.000 |
|  | 1st vs 3rd | -670.2 | 473 | 0.618 |
|  | 2nd vs 3rd | -596.5 | 473 | 0.716 |
| MF | Pre vs Injury | 964.2 | 166 | <0.001* |
|  | Pre vs 1st | 509.2 | 175 | 0.031* |
|  | Pre vs 2nd | 172.9 | 172 | 0.854 |
|  | Pre vs 3rd | 404.7 | 181 | 0.166 |
|  | Injury vs 1st | -455.0 | 175 | 0.072 |
|  | Injury vs 2nd | -791.4 | 173 | <0.001* |
|  | Injury vs 3rd | -559.5 | 181 | 0.018* |
|  | 1st vs 2nd | -336.4 | 180 | 0.335 |
|  | 1st vs 3rd | -104.5 | 188 | 0.981 |
|  | 2nd vs 3rd | 231.9 | 185 | 0.721 |

Pairwise comparisons were based on estimated marginal means from the linear mixed-effects model.

P values were adjusted for multiple comparisons using the Tukey method.

* P value < 0.05

SE, standard error; DF, defender; MF, midfielder; FW, forward; GK, goalkeeper.

**Supplementary Table S9. Distribution of ACL Injuries During Matches and Training by Position (Raw data)**

| Total  (n=488) | GK  (n=18) | DF  (n=160) | MF  (n=197) | FW  (n=113) | P value |
| --- | --- | --- | --- | --- | --- |
| Training | 6 (33.3) | 36 (22.5) | 40 (20.3) | 27 (23.9) | 0.597 |
| Game | 12 (66.7) | 124 (77.5) | 157 (79.7) | 86 (76.1) |  |
|  |  |  |  |  |  |
| Pro |  |  |  |  |  |
| Training | 5 (50.0) | 21 (35.0) | 16 (33.3) | 18 (52.9) | 0.230 |
| Game | 5 (50.0) | 39 (65.0) | 32 (66.7) | 16 (47.1) |  |
|  |  |  |  |  |  |
| Semi-professional |  |  |  |  |  |
| Training | 1 (20.0) | 12 (22.2) | 17 (19.8) | 5 (10.6) | 0.468 |
| Game | 4 (80.0) | 42 (77.8) | 69 (80.2) | 42 (89.4) |  |
|  |  |  |  |  |  |
| Amateur |  |  |  |  |  |
| Training | 0 (0.0) | 3 (6.5) | 7 (11.1) | 4 (12.5) | 0.734 |
| Game | 3 (100.0) | 43 (93.5) | 56 (88.9) | 28 (87.5) |  |

Values are presented as n (%).

P values were calculated using chi-squared tests to compare distributions between positions within each group.

GK, goalkeeper; DF, defender; MF, midfielder; FW, forward.

**Supplementary Table S10. Distribution of ACL Injuries During Matches and Training by Position (MI data)**

| Position | OR (95% CI) | p-value |
| --- | --- | --- |
| FW | 0.89 (0.51, 1.54) | 0.67 |
| GK | 0.59 (0.22, 1.60) | 0.30 |
| MF | 1.08 (0.66, 1.76) | 0.77 |

MI was used to handle missing data. ORs are relative to DF.

DF, defender; MF, midfielder; FW, forward; GK, goalkeeper; OR, odds ratio; CI, confidence intervals; MI, multivariate imputation.

**Supplementary Figure S1. Proportion of ACL Injuries during Matches and Training by Position (Raw data vs. MI data)**

Bar heights show the proportion (%) of injuries during matches and training by position. MI data reflect estimates after imputing missing values. Overall trends are similar between raw data and MI data.

GK, goalkeeper; DF, defender; MF, midfielder; FW, forward; MI, multivariate imputation.

**Supplementary Table S11. Distribution of ACL Injury Mechanisms during Matches by Position (Raw data)**

| Total  (n=274) | GK  (n=7) | DF  (n=91) | MF  (n=115) | FW  (n=61) | P value |
| --- | --- | --- | --- | --- | --- |
| direct | 2 (28.6) | 25 (27.5) | 27 (23.5) | 10 (16.4) | 0.321 |
| indirect | 2 (28.6) | 23 (25.3) | 24 (20.9) | 21 (34.4) |  |
| non-contact | 3 (42.9) | 37 (40.7) | 48 (41.7) | 27 (44.3) |  |
| unknown | 0 (0.0) | 6 (6.6) | 16 (13.9) | 3 (4.9) |  |
|  |  |  |  |  |  |
|  |  |  |  |  |  |
| Professional  (n=61) | GK  (n=3) | DF  (n=26) | MF  (n=23) | FW  (n=9) |  |
| direct | 1 (33.3) | 12 (46.2) | 8 (34.8) | 6 (66.7) | 0.430 |
| indirect | 1 (33.3) | 5 (19.2) | 3 (13.0) | 0 (0.0) |  |
| non-contact | 1 (33.3) | 9 (34.6) | 9 (39.1) | 3 (33.3) |  |
| unknown | 0 (0.0) | 0 (0.0) | 3 (13.0) | 0 (0.0) |  |
|  |  |  |  |  |  |
|  |  |  |  |  |  |
| Semi-professional  (n=115) | GK  (n=2) | DF  (n=30) | MF  (n=54) | FW  (n=29) |  |
| direct | 1 (50.0) | 2 (6.7) | 14 (25.9) | 2 (6.9) | 0.041* |
| indirect | 0 (0.0) | 11 (36.7) | 12 (22.2) | 15 (51.7) |  |
| non-contact | 1 (50.0) | 11 (36.7) | 18 (33.3) | 11 (37.9) |  |
| unknown | 0 (0.0) | 6 (20.0) | 10 (18.5) | 1 (3.4) |  |
|  |  |  |  |  |  |
|  |  |  |  |  |  |
| Amateur  (n=98) | GK  (n=2) | DF  (n=35) | MF  (n=38) | FW  (n=23) |  |
| direct | 0 (0.0) | 11 (31.4) | 5 (13.2) | 2 (8.7) | 0.395 |
| indirect | 1 (50.0) | 7 (20.0) | 9 (23.7) | 6 (26.1) |  |
| non-contact | 1 (50.0) | 17 (48.6) | 21 (55.3) | 13 (56.5) |  |
| unknown | 0 (0.0) | 0 (0.0) | 3 (7.9) | 2 (8.7) |  |

Values are presented as n (%).

P values were calculated using chi-squared tests to compare distributions between positions within each group. MFs had a higher proportion of direct injuries than FWs in Semi-professional players (p = 0.046, Holm-adjusted).

* P value < 0.05

GK, goalkeeper; DF, defender; MF, midfielder; FW, forward.

**Supplementary Table S12. Distribution of ACL injury mechanisms by position (MI data)**

| Mechanism | Position | OR (95% CI) | p-value |
| --- | --- | --- | --- |
| direct | FW | 0.47 (0.21, 1.01) | 0.06 |
|  | GK | 1.20 (0.25, 5.88) | 0.82 |
|  | MF | 0.72 (0.39, 1.32) | 0.29 |
| indirect | FW | 1.56 (0.79, 3.07) | 0.20 |
|  | GK | 1.19 (0.24, 5.78) | 0.83 |
|  | MF | 0.76 (0.40, 1.43) | 0.39 |
| non-contact | FW | 1.29 (0.68, 2.44) | 0.44 |
|  | GK | 0.91 (0.21, 4.00) | 0.90 |
|  | MF | 1.21 (0.71, 2.08) | 0.48 |
| unknown | FW | 0.67 (0.17, 2.66) | 0.57 |
|  | GK | 0.00 (0.00, Inf) | 0.99 |
|  | MF | 1.91 (0.75, 4.86) | 0.18 |

MI was used to handle missing data. ORs are relative to DF.

0.00–Inf CIs reflect few events.

DF, defender; MF, midfielder; FW, forward; GK, goalkeeper; OR, odds ratio; CI, confidence intervals; MI, multivariate imputation.

**Supplementary Figure S2. Proportion of Injury Mechanisms by Position (Raw data vs. MI data)**

Bar heights show the proportion (%) of injuries by player position. Unknown mechanisms were excluded. MI data reflect estimates after imputing missing values. Overall trends are similar between raw data and MI data.

GK, goalkeeper; DF, defender; MF, midfielder; FW, forward; MI, multivariate imputation.

**Supplementary Table S13. Distribution of Injury Action by Position (Raw data)**

| Total  (n=243) | GK  (n=6) | DF  (n=81) | MF  (n=100) | FW  (n=56) | P value |
| --- | --- | --- | --- | --- | --- |
| Ball reception | 0 (0) | 5 (6.2) | 14 (14.0) | 4 (7.1) | 0.002* |
| Pass | 0 (0) | 5 (6.2) | 3 (3.0) | 1 (1.8) |  |
| Cross | 0 (0) | 0 (0) | 1 (1.0) | 4 (7.1) |  |
| Shot | 0 (0) | 1 (1.2) | 7 (7.0) | 7 (12.5) |  |
| Sliding tackle | 0 (0) | 5 (6.2) | 2 (2.0) | 0 (0) |  |
| Heading | 0 (0) | 10 (12.3) | 7 (7.0) | 3 (5.4) |  |
| Tackle | 2 (33.3) | 30 (37.0) | 25 (25.0) | 15 (26.8) |  |
| Dribbling | 0 (0) | 5 (6.2) | 21 (21.0) | 11 (19.6) |  |
| Other | 4 (66.7) | 20 (24.7) | 20 (20.0) | 11 (19.6) |  |
|  |  |  |  |  |  |
| Professional  (n=58) | GK  (n=3) | DF  (n=24) | MF  (n=22) | FW  (n=9) |  |
| Ball reception | 0 (0) | 1 (5.6) | 2 (9.1) | 0 (0) | 0.229 |
| Pass | 0 (0) | 1 (5.6) | 1 (4.5) | 0 (0) |  |
| Shot | 0 (0) | 1 (5.6) | 1 (4.5) | 1 (11.1) |  |
| Sliding tackle | 0 (0) | 1 (5.6) | 0 (0) | 0 (0) |  |
| Heading | 0 (0) | 4 (22.2) | 3 (13.6) | 1 (11.1) |  |
| Tackle | 0 (0) | 7 (38.9) | 3 (13.6) | 2 (22.2) |  |
| Dribbling | 0 (0) | 2 (11.1) | 6 (27.3) | 0 (0) |  |
| Other | 3 (100) | 7 (38.9) | 6 (27.3) | 5 (55.6) |  |
|  |  |  |  |  |  |
| Semi-professional  (n=94) | GK  (n=1) | DF  (n=25) | MF  (n=43) | FW  (n=25) |  |
| Ball reception | 0 (0) | 3 (12.0) | 6 (13.8) | 4 (16.0) | 0.598 |
| Pass | 0 (0) | 3 (12.0) | 2 (4.6) | 1 (4.0) |  |
| Cross | 0 (0) | 0 (0) | 1 (2.3) | 2 (8.0) |  |
| Shot | 0 (0) | 0 (0) | 2 (4.6) | 3 (12.0) |  |
| Sliding tackle | 0 (0) | 1 (4.0) | 2 (4.6) | 0 (0) |  |
| Heading | 0 (0) | 3 (12.0) | 1 (2.3) | 1 (4.0) |  |
| Tackle | 1 (100) | 9 (36.0) | 9 (20.7) | 7 (28.0) |  |
| Dribbling | 0 (0) | 1 (4.0) | 9 (20.7) | 5 (20.0) |  |
| Other | 0 (0) | 5 (20.0) | 11 (25.3) | 2 (8.0) |  |
|  |  |  |  |  |  |
| Amateur  (n=90) | GK  (n=1) | DF  (n=32) | MF  (n=35) | FW  (n=22) |  |
| Ball reception | 0 (0) | 1 (3.1) | 6 (17.1) | 0 (0) | 0.117 |
| Pass | 0 (0) | 1 (3.1) | 0 (0) | 0 (0) |  |
| Cross | 0 (0) | 0 (0) | 0 (0) | 2 (9.1) |  |
| Shot | 0 (0) | 0 (0) | 4 (11.4) | 3 (13.6) |  |
| Sliding tackle | 0 (0) | 3 (9.4) | 0 (0) | 0 (0) |  |
| Heading | 0 (0) | 3 (9.4) | 3 (8.6) | 1 (4.5) |  |
| Tackle | 0 (0) | 14 (43.8) | 13 (37.1) | 6 (27.3) |  |
| Dribbling | 0 (0) | 2 (6.3) | 6 (17.1) | 6 (27.3) |  |
| Other | 1 (100) | 8 (25.0) | 3 (8.6) | 4 (18.2) |  |

Data are expressed as n (%) within each position.

* P value < 0.05

In total, the P value is 0.002, but not significant after Holm correction.

DF, defender; MF, midfielder; FW, forward; GK, goalkeeper.

**Supplementary Table S14. Distribution of ACL Injury Actions by Position (MI data)**

| Action | Position | OR (95% CI) | p-value |
| --- | --- | --- | --- |
| Ball reception | FW | 1.30 (0.36, 4.64) | 0.69 |
|  | GK | 0.00 (0.00, Inf) | 0.99 |
|  | MF | 2.65 (0.98, 7.20) | 0.06 |
| Pass | FW | 0.42 (0.06, 2.93) | 0.38 |
|  | GK | 0.00 (0.00, Inf) | 0.99 |
|  | MF | 0.60 (0.15, 2.37) | 0.47 |
| Cross | FW | 344588.87 (0.00, Inf) | 0.99 |
|  | GK | 1.88 (0.00, Inf) | 1.00 |
|  | MF | 65574.03 (0.00, Inf) | 0.99 |
| Shot | FW | 1.00 (0.00, Inf) | 1.00 |
|  | GK | 1.00 (0.00, Inf) | 1.00 |
|  | MF | 1.00 (0.00, Inf) | 1.00 |
| Sliding tackle | FW | 1.00 (0.00, Inf) | 1.00 |
|  | GK | 1.00 (0.00, Inf) | 1.00 |
|  | MF | 1.00 (0.00, Inf) | 1.00 |
| Heading | FW | 0.42 (0.13, 1.42) | 0.17 |
|  | GK | 0.00 (0.00, Inf) | 0.99 |
|  | MF | 0.45 (0.18, 1.14) | 0.09 |
| Tackle | FW | 0.67 (0.34, 1.33) | 0.26 |
|  | GK | 0.68 (0.11, 4.03) | 0.67 |
|  | MF | 0.63 (0.34–, 1.14) | 0.13 |
| Dribbling | FW | 3.70 (1.27, 10.75) | 0.02 |
|  | GK | 0.00 (0.00, Inf) | 0.98 |
|  | MF | 4.18 (1.58, 11.08) | <0.001* |
| Other | FW | 0.68 (0.30, 1.50) | 0.34 |
|  | GK | 1.17 (0.23, 6.09) | 0.85 |
|  | MF | 0.70 (0.36, 1.36) | 0.30 |

MI was used to handle missing data. ORs are relative to DF.

Extremely large ORs and 0.00–Inf CIs reflect few events.

* P value < 0.05

DF, defender; MF, midfielder; FW, forward; GK, goalkeeper; OR, odds ratio; CI, confidence intervals; MI, multivariate imputation.

**Supplementary Figure S3. Proportion of Injury Actions by Position (Raw data vs. MI data)**
Bar heights show the proportion (%) of injuries by player position. Injuries categorized as "Other" are excluded. MI data reflect estimates after imputing missing values. Overall trends are similar between raw data and MI data. However, for GKs the numbers were small and several actions were missing, resulting in large difference from MI estimates.
GK, goalkeeper; DF, defender; MF, midfielder; FW, forward; MI, multivariate imputation.
